# Combination of Ascorbic Acid and Menadione Induces Cytotoxic Autophagy in Human Glioblastoma Cells

**DOI:** 10.1155/2022/2998132

**Published:** 2022-03-23

**Authors:** Ana Despotović, Aleksandar Mirčić, Sonja Misirlić-Denčić, Ljubica Harhaji-Trajković, Vladimir Trajković, Nevena Zogović, Gordana Tovilović-Kovačević

**Affiliations:** ^1^Department of Neurophysiology, Institute for Biological Research “Siniša Stanković”–National Institute of Republic of Serbia, University of Belgrade, Bulevar despota Stefana 142, 11000 Belgrade, Serbia; ^2^Institute of Histology and Embryology, Faculty of Medicine, University of Belgrade, Višegradska 26, 11000 Belgrade, Serbia; ^3^Institute of Medical and Clinical Biochemistry, Faculty of Medicine, University of Belgrade, Pasterova 2, 11000 Belgrade, Serbia; ^4^Institute of Microbiology and Immunology, Faculty of Medicine, University of Belgrade, Dr. Subotića 1, 11000 Belgrade, Serbia; ^5^Department of Biochemistry, Institute for Biological Research “Siniša Stanković”–National Institute of Republic of Serbia, University of Belgrade, Bulevar despota Stefana 142, 11000 Belgrade, Serbia

## Abstract

We investigated the ability of the ascorbic acid (AA) and menadione (MD) combination, the well-known reactive oxidative species- (ROS-) generating system, to induce autophagy in human U251 glioblastoma cells. A combination of AA and MD (AA+MD), in contrast to single treatments, induced necrosis-like cell death mediated by mitochondrial membrane depolarization and extremely high oxidative stress. AA+MD, and to a lesser extent MD alone, prompted the appearance of autophagy markers such as autophagic vacuoles, autophagosome-associated LC3-II protein, degradation of p62, and increased expression of beclin-1. While both MD and AA+MD increased phosphorylation of AMP-activated protein kinase (AMPK), the well-known autophagy promotor, only the combined treatment affected its downstream targets, mechanistic target of rapamycin complex 1 (mTORC1), Unc 51-like kinase 1 (ULK1), and increased the expression of several autophagy-related genes. Antioxidant N-acetyl cysteine reduced both MD- and AA+MD-induced autophagy, as well as changes in AMPK/mTORC1/ULK1 activity and cell death triggered by the drug combination. Pharmacological and genetic autophagy silencing abolished the toxicity of AA+MD, while autophagy upregulation enhanced the toxicity of both AA+MD and MD. Therefore, by upregulating oxidative stress, inhibiting mTORC1, and activating ULK1, AA converts MD-induced AMPK-dependent autophagy from nontoxic to cytotoxic. These results suggest that AA+MD or MD treatment in combination with autophagy inducers could be further investigated as a novel approach for glioblastoma therapy.

## 1. Introduction

Glioblastoma multiforme (GBM) represents the most prevalent and aggressive brain tumor in the adult population [1]. The standard care for GBM includes maximal resection, followed by radiation therapy given concomitantly with the alkylating agent temozolomide. Nonetheless, the average survival time for GMB patients is approximately 10 months. The development of new, more efficient treatment strategies remains challenging due to limited drug entry into the central nervous system, the highly infiltrative nature of the tumor, and the resistance of stem-like tumor cells to chemotherapy, radiotherapy, and immunotherapy [2].

Menadione (2-methyl-1,4-naphthoquinone; MD, vitamin K3) is a lipid-soluble synthetic analog of vitamin K, metabolized by the human body into menaquinone (vitamin K2). Numerous studies showed potent anticancer activity of MD in various cancer cell lines, with both antiproliferative and direct cytotoxic action resulting in caspase-dependent [3–6], caspase-independent apoptosis [7], necroptosis [8], or necrosis-like cell death [9]. The main mechanism of MD cytotoxic action is closely related to reactive oxygen species (ROS) produced during its metabolism. Paradoxically, a well-known antioxidant ascorbic acid (AA) is found to augment MD-induced ROS accumulation by stimulating electron transfer from MD to molecular oxygen, thereby enhancing the cytotoxicity of MD [10]. Similarly to the toxicity of MD alone, several types of AA+MD-induced cancer cell death have been proposed, including caspase- or AIF-dependent apoptosis [11, 12], PARP-dependent [10] and PARP-independent necrosis-like death [13], or autoschizis, a specific form of cell death caused by AA+MD [14, 15]. Potent antitumor activity of AA+MD is also observed in various animal models of cancer [16, 17]. Oral administration of AA+MD (APATONE®) showed promise in delaying progression of end-stage prostate cancer in a clinical trial [18], and the drug has received Orphan Drug Designation for treating advanced inoperable bladder cancer (stage III and IV) [19].

Macroautophagy (hereafter autophagy) represents a lysosome-dependent cellular degradation program for the removal of superfluous and dysfunctional organelles and proteins. Cytoplasmic components and organelles are sequestered within autophagosomes that subsequently fuse with lysosomes into autolysosomes, where the engulfed content is degraded and recycled. During the process of sequestration, a lipidated form of microtubule-associated protein 1 light chain 3 protein (LC3-II) is formed and recruited to the autophagosomal membrane. Therefore, the amount of LC3-II protein in the cell serves as an early autophagy marker. Molecules and cellular components selectively targeted for autophagic degradation are labeled by p62 (sequestosome 1, SQSTM1/p62) and delivered to autophagosomes through an LC3-interacting region of p62. Subsequent degradation of this cargo receptor and specific autophagy substrate in the autolysosomes represents another important determinant of the ongoing autophagic process. The autophagy pathway is tightly controlled by the serine/threonine kinase activity of the main autophagy repressor, mechanistic target of rapamycin complex 1 (mTORC1). mTORC1 inhibits autophagy during nutrient-rich conditions by suppressing the activity of the autophagosome biogenesis-initiating complex consisting of Unc-51-like autophagy-activating kinase 1 (ULK1), autophagy-related 13 (Atg13), and focal adhesion kinase family interacting protein of 200 kD [20]. However, during metabolic/energy stress, the high AMP/ATP ratio in the cell is sensed by a positive autophagy regulator, AMP-activated protein kinase. AMPK promotes autophagy either by indirect inhibition of mTORC1 through phosphorylation of mTOR-adapter proteins RAPTOR (regulatory-associated protein of mTOR) and TSC2 (tuberous sclerosis complex 2), or by direct activation of ULK1 (phosphorylation at Ser317) and beclin-1, the key regulator of autophagosome formation [20]. Autophagy could be also modulated at the transcription level by transcription factor EB (TFEB) and the forkhead transcription factors (FOXO). TFEB and FOXO regulate transcription of different autophagy-related genes, while TFEB promotes the transcription of several genes responsible for lysosomal biogenesis and function [21]. Their activity is regulated through phosphorylation and subsequent nuclear translocation inhibition, which in turn blocks autophagy-related gene expression and autophagy. The main kinase responsible for TFEB phosphorylation in the presence of amino acids is mTOR [22, 23], while FOXO is predominantly modulated by AKT in response to growth factors and insulin stimulation [21]. Once activated, autophagy can have a dual role in the cell, it could promote cell adaptation and survival in response to stress conditions, or it may act as an alternative route of cell death when designated as cytotoxic [24].

The ability of both MD [25–32] and combination of MD with AA [15, 33] to induce autophagy has previously been demonstrated. However, there are conflicting data about the role of MD- and AA+MD-induced autophagy in cell survival and death in diferent cells/experimental settings. While MD induced a prosurvival autophagic response in HeLa human cervical carcinoma cell line, U937 human histiocytic lymphoma cells, and immortalized rat hepatocytes [25, 26, 31], MD-triggered autophagy was toxic to C2C12 mouse myoblasts [29]. Autophagy induced by the combination of AA and MD was cytoprotective in PC3 human prostate carcinoma cell line [33] but had no effect on the survival of MCF7 human breast carcinoma cells [10]. However, the molecular mechanisms of autophagy induction by AA+MD combination and its interaction with oxidative stress have not been evaluated. It should also be noted that the role of AA+MD-induced autophagy in cell survival/death was assessed only using pharmacological inhibitors of autophagy [10, 33], which are known for their autophagy-independent effects on cell viability [34–36]. Finally, the effect of AA+MD combination on autophagy induction in human U251 glioblastoma cells, as well as its possible influence on their survival, has not been investigated so far.

The present study demonstrates, for the first time, that the treatment of U251 human glioblastoma cells with AA increased MD-mediated AMPK-dependent autophagy through oxidative stress-dependent mTORC1 inhibition and subsequent ULK1 activation. Moreover, using both pharmacological and genetic approaches, we show that, in contrast to mild nontoxic autophagy triggered by MD, its increase by AA cotreatment contributed to the necrotic demise of glioblastoma cells.

## 2. Material and Methods

### 2.1. Chemicals

Ascorbic acid, menadione, Z-VAD, Q-VAD, staurosporine (STS), 3-methyladenine (3MA), ammonium chloride (NH_4_Cl), bafilomycin A1 (BAF), trehalose (TREH), and N-acetylcysteine (NAC) are all purchased from Sigma-Aldrich (St. Louis, MO, USA).

### 2.2. Equipment

The absorbance of colored substrates (CV and MTT) was measured in an automated microplate reader (Sunrise, Tecan, Dorset, UK). Flow cytometric analyses of apoptosis/necrosis, caspase activation, mitochondrial membrane potential, total intracellular production of ROS, mitochondrial superoxide, and intracellular acidic vesicles were conducted using FACSCalibur flow cytometer equipped with CellQuest Pro software (BD Biosciences, Heidelberg, Germany). SDS-PAGE electrophoresis was performed on Hoefer's (Holliston, MA, USA) standard vertical protein electrophoresis unit, while proteins were transferred to nitrocellulose membranes using Bio-Rad's (Hercules, CA, USA) transfer unit. RT-qPCR analysis was performed on a Realplex2 Mastercycler (Eppendorf, Hamburg, Germany). Light microscopy was conducted using an universal inverted microscope (Leica Microsystems DMIL, Wetzlar, Germany) equipped with Leica Microsystems DFC320 camera, while ultrastructural morphology of cells was analysed by Morgagni 268D electron microscope (FEI, Hillsboro, OR, USA) using a MegaView III CCD camera equipped with iTEM software (Olympus Soft Imaging Solutions, Münster, Germany).

### 2.3. Cell Culture

The human glioblastoma cell line U251 was obtained from the European Collection of Animal Cell Cultures (Salisbury, UK). The cells were incubated at 37°C, in a humidified atmosphere with 5% CO_2_, in the RPMI cell culture medium (Gibco, Life Technologies, MA, USA) supplemented with 5% fetal calf serum (FCS), 2 mM L-glutamine, 10 mM sodium pyruvate, and penicillin/streptomycin (all from Capricorn Scientific, Ebsdorfergrund, Germany). Cells were prepared for experiments using the conventional trypsinization procedure with trypsin/EDTA and seeded in 96-well flat-bottom plates (1 × 10^4^ cells/well) for the $\underset{¯}{\text{cell viability}}$ assessment, 6-well plates (2.5 × 10^5^ cells/well) for flow cytometric analysis, or 60 mm Petri dishes (1 × 10^6^ cells) for the electron microscopy, immunoblot, and reverse transcription quantitative real-time polymerase chain reaction (RT-qPCR).

### 2.4. Treatments

The cells were rested in a cell culture medium for 24 h and then treated with AA, MD, and their combination, in the presence or absence of caspase inhibitors (Z-VAD and Q-VAD), apoptosis inducer STS, autophagy inhibitors 3MA, NH_4_Cl, and BAF, autophagy inducer TREH, or antioxidant NAC as described in figure legends.

### 2.5. Cell Viability

#### 2.5.1. CV and MTT Assays

The number of adherent cells and mitochondrial dehydrogenase activity, as the indicators of cell viability, was assessed by crystal violet (CV) and 3-(4,5-dimethylthiazol-2-yl)-2,5-diphenyl-tetrazolium bromide (MTT) reduction assay, respectively.

For the CV test, cell cultures were washed with phosphate buffer saline (PBS) to remove nonadherent dead cells. The adherent, viable cells were fixed with methanol and stained with 10% CV solution (Sigma-Aldrich, St. Louis, MO, USA) at room temperature for 15 minutes. After rigorous washing with water, CV was dissolved in 33% acetic acid. The absorbance of dissolved dye, corresponding to the number of adherent (viable) cells, was measured in an automated microplate reader at 570 nm (Sunrise, Tecan, Dorset, UK).

For the MTT assay, cells were washed with PBS to avoid nonspecific reaction of MTT with AA, MTT (Sigma-Aldrich, St. Louis, MO, USA) solution was added at a final concentration of 0.5 mg/ml, and cultures were incubated for an additional hour. The solution was then removed, and cells were lysed with DMSO (Sigma-Aldrich, St. Louis, MO, USA). The absorbance of the purple-colored formazan, which is in direct proportion to the number of metabolically active cells, was monitored by the automated microplate reader at 570 nm (Sunrise, Tecan, Dorset, UK). The results of both assays were presented as % viability relative to untreated control cultures, taken as 100% viable.

### 2.6. Mathematical Analysis of Synergism

To describe the type of interaction between ascorbate and menadione, cells were treated with each agent alone and their appropriate combinations. The number of viable cells was determined by the CV assay. The values of the combination index (CI) reflecting synergistic (CI < 1), additive (CI = 1), or antagonistic interactions (CI > 1) were calculated according to the method designed by Chou and Talalay [37] using CompuSyn software (https://www.combosyn.com).

### 2.7. FACS Analyses

#### 2.7.1. Analysis of Apoptosis and Necrosis

Apoptotic and necrotic cell death was analyzed by double staining with fluorescein isothiocyanate- (FITC-) conjugated annexin V (BD Biosciences, Heidelberg, Germany) and propidium iodide (PI, Sigma-Aldrich, St. Louis, MO, USA). Annexin V-FITC binds to phosphatidylserine exposed on the external surface of the cell in the early stage of apoptosis, while PI is a cell impermeable DNA-intercalating dye that labels cells with the damaged cell membrane. Cells were detached by trypsinization, stained with annexin V-FITC (2 *μ*g/ml) and PI (20 *μ*g/ml), and incubated in the dark for 15 minutes at 37°C. The green (FL1) and red (FL2) fluorescence was measured using FACSCalibur flow cytometer equipped with CellQuest Pro software (BD Biosciences, Heidelberg, Germany) to evaluate the number of viable (annexin^−^/PI^−^), early apoptotic (annexin^+^/PI^−^), and late apoptotic/necrotic cells (annexin^+^/PI^+^).

#### 2.7.2. Caspase Activation

Activation of caspases, the cysteine proteases involved in the execution of apoptotic cell death, was measured by flow cytometry after staining the cells with a cell-permeable, FITC-conjugated pancaspase inhibitor ApoStat (R&D Systems, Minneapolis, MN, USA). Cells were detached by trypsinization and stained with ApoStat (0.5 *μ*g/ml) for 30 minutes at 37°C. The increase in green fluorescence (FL1) as a measure of caspase activity was determined using FACSCalibur flow cytometer equipped with Cell Quest Pro software. The results were expressed as the % of cells containing active caspases.

#### 2.7.3. Mitochondrial Membrane Potential Assessment

Mitochondrial membrane potential was assessed using JC-1 (5,5′,6,6′-tetrachloro1,1′,3,3′-tetraethylbenzimidazolocarbocyanine iodide; Sigma-Aldrich, St. Louis, MO, USA). This lipophilic cation, capable of entering selectively into mitochondria, has the property of altering aggregation status depending on mitochondrial membrane potential—it changes reversibly from green monomeric form to orange/red aggregates as the membrane potential increases. Cells were detached by trypsinization and stained with JC-1 (5 *μ*g/ml in a JC-1 staining buffer) for 20 minutes at 37°C. The green monomers and red aggregates were detected by FACSCalibur flow cytometer and analyzed using CellQuest Pro software. The results were presented as the fold change in red/green fluorescence ratio (mean FL2/FL1, arbitrarily set to 1 in control samples), with the decrease in FL2/FL1 reflecting mitochondrial depolarization.

#### 2.7.4. ROS Measurement

The total intracellular production of ROS and mitochondrial superoxide generation were determined using dihydrorhodamine 123 (DHR) and MitoSOX Red (both from Thermo Fisher Scientific, Waltham, MA), respectively, as previously described [38]. DHR is a cell-permeable, nonfluorescent ROS indicator that is oxidized by ROS to its green-fluorescent derivative rhodamine 123. MitoSOX Red enters into living cells and selectively targets mitochondria where it fluoresces red when oxidized by superoxide. DHR (2 *μ*M) was added to the cell cultures at the beginning of treatment, while MitoSOX Red (5 *μ*M) was added during the last 10 minutes of treatment. At the end of incubation, the cells were detached by trypsinization and washed in PBS. The mean intensity of green (FL1) or red (FL2) fluorescence, corresponding to total ROS production or mitochondrial superoxide generation, respectively, was determined using a FACSCalibur flow cytometer and CellQuest Pro software. The results were presented as the fold change relative to the FL1 (DHR) or FL2 (MitoSOX Red) value of the untreated cells, which was arbitrarily set to 1.

#### 2.7.5. Detection of Acidic Intracellular Vesicles

The intracellular acidic vesicles (i.e., lysosomes and autophagolysomes) were stained with LysoTracker Red DND-99 (LTR; Thermo Fisher Scientific, Waltham, MA, USA) and analyzed by flow cytometry. LTR consists of a red fluorophore DND-99 linked to a weak base that is only partially protonated at neutral pH, allowing LTR to freely permeate cell membranes and selectively accumulate in acidic vesicles due to their low pH. The cells were incubated with LTR (0.1 *μ*M) for 1 hour at 37°C, washed in PBS, trypsinized, and analyzed using a FACSCalibur flow cytometer and CellQuest Pro software. The results were presented as a fold increase in the mean fluorescence intensity relative to the FL2 value obtained in untreated cells, which was arbitrarily set to 1.

### 2.8. Immunoblotting

Immunoblotting was used to assess the expression/activation of specific proteins. At the end of treatment, the dead cells were removed with cold PBS and the remaining adherent cells were lysed in lysis buffer (30 mM Tris–HCl pH 8.0, 150 mM NaCl, 1% Nonidet P-40, 1 mM PMSF, and protease/phosphatase inhibitor mixture; all from Sigma-Aldrich, St. Louis, MO, USA) on ice for 30 minutes. The cell lysates were centrifuged at 14000*g* for 15 minutes at 4°C, and the supernatants were collected. Equal amounts of proteins from each sample were separated by SDS-PAGE and transferred to nitrocellulose membranes (Bio-Rad, Hercules, CA, USA). Rabbit anti-human antibodies against microtubule-associated protein 1 light-chain 3B (LC3B), mTOR, phospho-mTOR (Ser2448), AMPK*α*, phospho-AMPK*α* (Thr-172), p70S6 kinase (S6K), phospho-S6K (Thr389), ULK1, phospho-ULK1 (Ser317), beclin-1, cleaved caspase 3, poly (ADP-ribose) polymerase-1 (PARP1), and *β*-actin (Cell Signaling Technology, Danvers, MA, USA), mouse anti-human antibodies against p62/sequestosome 1 (Novus Biologicals, Littleton, CO, USA), and TFEB (Santa Cruz Biotechnology, Dallas, TX, USA) were used as primary antibodies, whereas peroxidase-conjugated goat anti-rabbit IgG (Cell Signaling Technology, Danvers, MA) and goat anti-mouse IgG (Southern Biotech, Birmingham, AL, USA) were used as secondary antibodies. The specific protein bands were visualized by chemiluminescence using the Amersham reagent (Amersham Pharmacia Biotech, Piscataway, NJ, USA) on a ChemiDoc Imaging System (Bio-Rad Laboratories, Hercules, CA, USA). The signal intensity was quantified by densitometry using ImageJ software (https://imagej.nih.gov/ij/, National Institutes of Health, Bethesda, MA, USA), and the ratio between phosphorylated and corresponding total protein signals or actin (for LC3-II, p62, and beclin-1) was calculated. The results were presented relative to the signal intensity of the untreated control, which was arbitrarily set to 1.

### 2.9. RT-qPCR

RT-qPCR was used to determine the expression of ATG genes. Total RNA was extracted from cells with TRIzol Reagent (Invitrogen, Caarlsbad, CA, USA), and 1 *μ*g of RNA was used in the reverse transcription reaction using M-MuLV reverse transcriptase and random hexamer primers (all from Thermo Fisher Scientific, Waltham, MA, USA) according to the manufacturer's instructions. RT-qPCR analysis was performed in a Realplex^2^ Mastercycler (Eppendorf, Hamburg, Germany) using 96-well reaction plates (Applied Biosystems, Foster City, CA, USA), Maxima Hot start PCR Master Mix (Thermo Fisher Scientific, Waltham, MA, USA), and the TaqMan primers/probes for human ATG3 (Hs00223937_m1), ATG13 (Hs00207186_m1), forkhead box O1 (FOXO1; Hs01054576_m1), forkhead box O3F (FOXO3F, Hs00921424_m1), UV radiation resistance-associated gene (UVRAG; Hs00163433_m1), Bax-interacting factor 1/endophilin-B1 (BIF1; Hs00211220_m1), autophagy/beclin-1 regulator 1 (AMBRA1; Hs00387943_m1), *γ*-aminobutyric acid receptor-associated protein (GABARAP; Hs00925899_m1), phosphatidylinositol 3-kinase catalytic subunit type 3 (PIK3C3; Hs00176908_m1), beclin-1 (Hs00186838_m1), p62/sequestosome 1 (Hs00177654_m1), and 18S ribosomal RNA (RN18S; Hs03928985_g1) (all from Thermo Fisher Scientific, Waltham, MA, USA). The thermal cycle conditions were 95°C for 4 min, followed by 40 cycles of 15 s at 95°C and 1 min at 60°C. All reactions were performed in triplicates. The average cycle of threshold (Ct) values of 18 s RNA as housekeeping gene was subtracted from the Ct values of target genes to obtain ΔCt, and relative gene expression was determined as 2^−ΔCt^. The results were presented relative to the control value, which was arbitrarily set to 1.

### 2.10. Transmission Electron Microscopy (TEM)

For the analysis of ultrastructural morphology by TEM, cells were trypsinized, fixed in 3% glutaraldehyde in cacodylate buffer, postfixed in 1% osmium tetroxide, dehydrated in graded alcohols, and then embedded in epoxy medium (all from Sigma-Aldrich, St. Louis, MO, USA). The ultrathin sections were stained in uranyl acetate and lead citrate and examined using a Morgagni 268D electron microscope (FEI, Hillsboro, OR, USA). The images were acquired using a MegaView III CCD camera equipped with iTEM software (Olympus Soft Imaging Solutions, Münster, Germany).

### 2.11. RNA Interference (RNAi)

Small interfering RNA (siRNA) targeting human LC3 and beclin-1, as well as scrambled control siRNA, were obtained from Santa Cruz Biotechnology (Dallas, TX, USA). The empty control (pcDNA3) and transcription factor EB- (TFEB-) encoding plasmid vectors were kindly donated by Dr Viktor I. Korolchuk (Newcastle University, UK). The transfection of U251 cells with plasmids and siRNA was performed in 96-well microplates for cell viability assays, or in 6-well plates for flow cytometry, using Lipofectamine 2000 (Thermo Fisher Scientific, Waltham, MA, USA). The plasmids (10 ng/*μ*l) and siRNA oligomers (100 nM), as well as Lipofectamine 2000 (2.8 *μ*g/ml), were dissolved and incubated for 5 minutes in Opti-MEM medium without FCS and antibiotics. The equal amounts of medium with siRNA or plasmid and medium with Lipofectamine 2000 were mixed and incubated for 20 minutes, allowing the formation of RNA-liposome or plasmid-liposome complexes. Afterwards, the cells were incubated with siRNA/Lipofectamine 2000 or plasmid/Lipofectamine 2000 mixture for 8 h. Cells were rested for 24 h following transfection and then treated with AA and MD.

### 2.12. Statistical Analysis

Data were evaluated statistically by one-way ANOVA followed by Tukey's test for multiple comparisons. A *p* value of less than 0.05 was considered statistically significant.

## 3. Results

### 3.1. Combination of AA and MD Exerts Synergistic Antiglioblastoma Effect in U251 Cells

**Figure 1:**
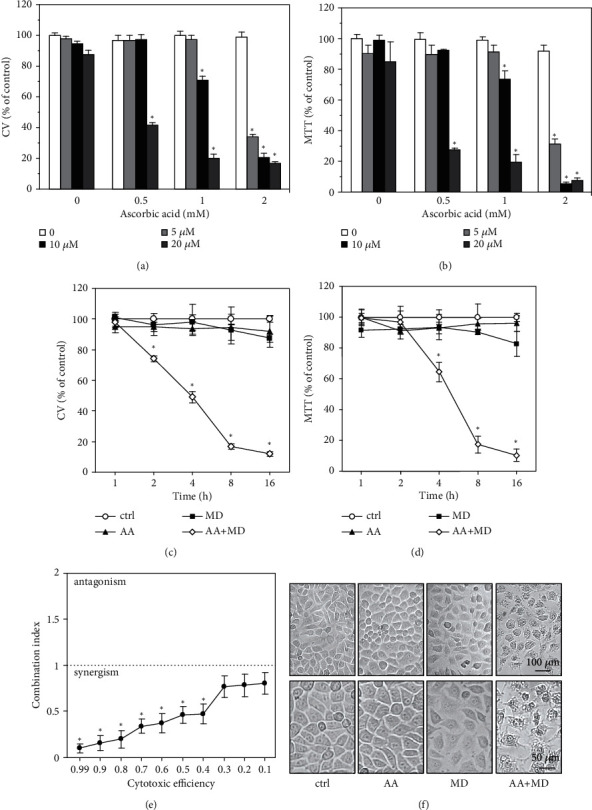
The combination of AA and MD exerts dose- and time-dependent cytotoxicity in U251 cells. U251 cells were incubated with different concentrations of AA (0.5–2 mM) and MD (5–20 *μ*M) alone and in combinations (AA+MD) for 24 h (a, b) or with AA (1 mM) and MD (20 *μ*M) and their combination for indicated time periods (c, d). Cell viability was assessed using crystal violet (CV) (a, c) or MTT tests (b, d), while cell morphology was analyzed using phase-contrast microscopy after 4 h (f). The combination index was calculated using Chou–Talalay method (e). The data are presented as mean ± standard deviation (SD) values of triplicates (a–e) or pictures (f) from one representative of three independent experiments. ^∗^*p* < 0.05 compared to untreated, control cells.

To determine the in vitro antiglioblastoma potential of AA+MD combination, U251 cells were exposed to AA (0–2 mM) and/or MD (5–20 *μ*M) for 24 h (Figures 1(a) and 1(b)). U251 cells were insensitive to AA applied alone, while MD at the highest concentration (20 *μ*M) only slightly decreased the number of adherent viable cells and mitochondrial dehydrogenase activity measured by CV (Figure 1(a)) and MTT test (Figure 1(b)), respectively. On the other hand, simultaneous treatment with AA and MD strongly reduced the viability of U251 cells in a dose-dependent manner (Figures 1(a) and 1(b)), indicating a strong synergistic interaction between MD and AA [39]. Indeed, this observation was confirmed by Chou-Talalay analysis (CI < 1) [37] (Figure 1(e)). The concentrations selected for further experiments were 1 mM of AA and 20 *μ*M of MD, as their combination caused more than 80% decrease in cell viability after only 8 h of incubation (Figures 1(c) and 1(d)). Accordingly, phase-contrast microscopy demonstrated that 4 h incubation with AA or MD alone had no significant effect on cell morphology, while combined treatment induced strong cytoplasmic vacuolization and a significant decrease in cell density (Figure 1(f)). Collectively, these data demonstrate a synergistic antiglioblastoma effect of AA and MD in vitro.

### 3.2. AA+MD Combination Induces ROS- and Mitochondrial Depolarization-Mediated Necrotic Cell Death

**Figure 2:**
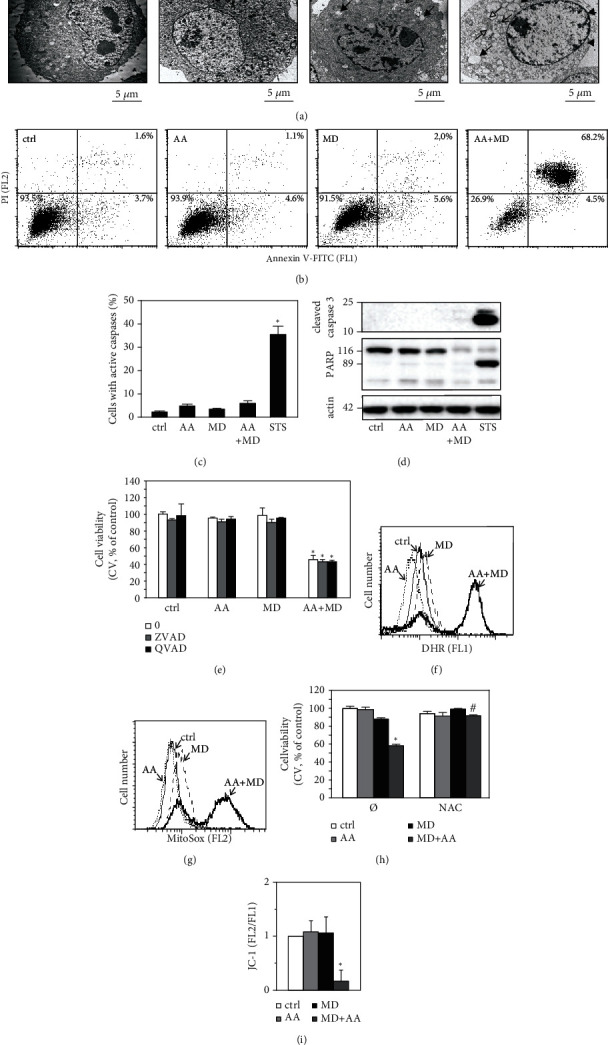
The combination of AA and MD induces ROS- and mitochondrial depolarization-mediated necrosis-like U251 cell death. U251 cells were treated with AA (1 mM), MD (20 *μ*M), and their combination (AA+MD) (a–i), or staurosporine (1 *μ*M, STS) (c, d) in absence or presence of N-acetyl cysteine (2 mM, NAC) (h) and ZVAD or QVAD (10 *μ*M) (e). Ultrastructural electron microscopy analysis was carried out after 8 h treatment; *arrowhead* indicates disintegrated plasma membrane, *full arrow* indicates vacuolization, *empty arrow* indicates damaged mitochondria with discontinued cristae, and *plain arrow* indicates autophagic-like vesicle containing cellular components (a). After 8 h (b) or 4 h (c, f, g, i), the cells were stained with AnnexinV-FITC/PI (b), ApoStat (c), DHR (f), MitoSox (g), and JC-1 (i), and fluorescence intensity indicating the presence of apoptotic/necrotic cells (b), caspase activation (c), intracellular ROS production (f), mitochondrial superoxide production (g), and mitochondrial depolarization (i) was measured using flow cytometry. The levels of cleaved caspase 3, PARP, and actin as a loading control were analyzed by immunoblotting (d), while the cell viability was measured using CV test (e, h) after 4 h treatment. The data presented as representative micrographs (a), dot plots (b), immunoblots (d), or histograms (f, g) are shown. The data are presented as mean ± SD values from three independent experiments (i, c) or mean ± SD values of triplicates from a representative of three independent experiments (e, h). ^∗^*p* < 0.05 compared to untreated, control cells; ^#^*p* < 0.05 compared to cells treated with AA+MD.

In the next set of experiments, we investigated the type and mechanisms of cell death induced by the combined treatment. Ultrastructural TEM analysis demonstrated no apparent morphological changes in cells treated with AA for 8 h (Figure 2(a)). In contrast, the cells exposed to MD displayed cytoplasmic vacuolization, but without any signs of cell death. Consistent with the cell viability data, morphological changes characteristic of necrotic cell death, such as disintegration of the plasma membrane, loss of electron density in the cytosol, extensive vacuolization, the presence of dilated mitochondria with hypodense matrix and discontinued cristae, and preserved cell nuclei were clearly visible in cells incubated with AA+MD combination (Figure 2(a)). Next, flow cytometric analysis of cells stained with Annexin V-FITC/PI demonstrated that single AA and MD treatments had no effect on the number of live/dead cells, while their combination strongly increased the proportion of necrotic Ann+/PI+ cells, but not apoptotic Ann+/PI- cells (Figure 2(b)). Furthermore, AA, MD, as well as their combination, failed to activate caspases (Figure 2(c)), including the main executioner caspase-3 (Figure 2(d)), and to induce PARP cleavage (Figure 2(d)), as demonstrated by flow cytometry of Apostat-stained cells (Figure 2(c)) and immunoblot (Figure 2(d)). Accordingly, caspase inhibitors Z-VAD and Q-VAD did not protect U251 cells from AA+MD-induced damage (Figure 2(e)), arguing against the involvement of caspase-dependent apoptosis in antiglioblastoma effect of combined treatment. On the other hand, a proapototic agent STS, used here as a positive control for apoptosis induction, markedly increased the number of cells with active caspases (Figure 2(c)), triggered cleavage of caspase-3, and prompted PARP activation (Figure 2(d)). In accordance with previous findings [8, 40], MD and especially the combined treatment induced an increase in total ROS and mitochondrial superoxide levels, as demonstrated by flow cytometric analysis of DHR- (Figure 2(f)) and MitoSOX-stained cells (Figure 2(g)), respectively. The well-known antioxidant NAC efficiently protected U251 cells from combined treatment (Figure 2(h)), indicating a pivotal role of oxidative stress in AA+MD-induced cytotoxicity. Finally, AA or MD alone had no significant effect on mitochondrial membrane potential, while their combination induced an intense mitochondrial depolarization, as shown by flow cytometric analysis of cells stained with JC-1 (Figure 2(i)). Taken together, these findings demonstrate that AA+MD combination induces oxidative stress- and mitochondrial depolarization-dependent necrotic death of U251 cells.

### 3.3. AA Enhances MD-Induced Autophagic Flux and Transcription of Autophagy Genes

**Figure 3:**
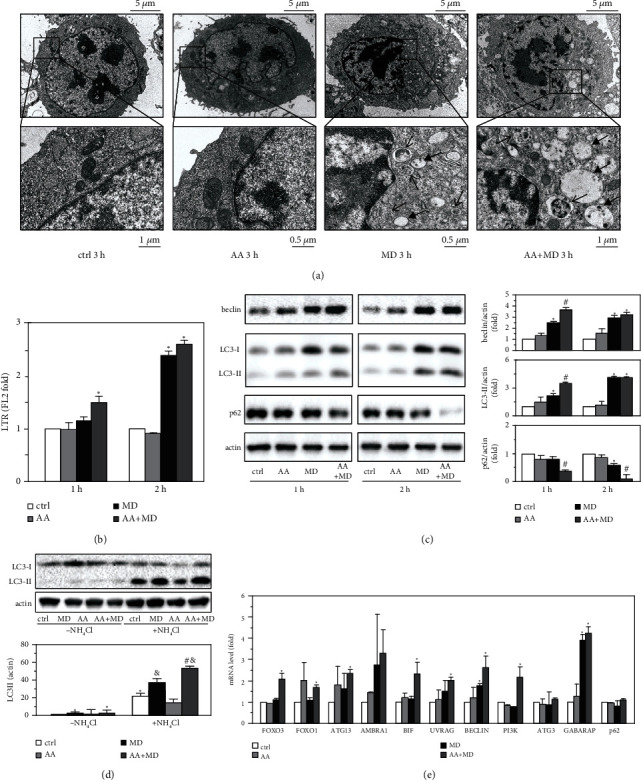
MD and AA+MD combination induce autophagy in U251 cells. U251 cells were treated with AA (1 mM), MD (20 *μ*M), and their combination (AA+MD) for indicated time periods (b, c), 3 h (a), 2 h (d), or 0.5 h (e), in the absence (a–c, e) or the presence (d) of proteolysis inhibitor NH_4_Cl (50 mM). Morphological changes were assessed using ultrastructural electron microscopy; *full arrow* indicates vacuolization and *plain arrow* indicates autophagic-like vesicle containing cellular components (a). The fluorescence of LTR-stained acidic intracellular vesicles was detected by using flow cytometry (b). The levels of beclin-1, p62 (c), and LC3I/II (c, d) (relative to actin) were determined by immunoblotting and quantified using densitometry, while the expression of autophagy-related genes was analyzed by RT-qPCR (e). The results are presented as representative micrographs (a), mean ± SD values from three independent (b–d), or one representative of three independent experiments (e). ^∗^*p* < 0.05 compared to untreated, control cells arbitrarily set to 1 (b–e); ^#^*p* < 0.05 compared to treatment with MD (c) or NH_4_Cl + MD (d); ^&^*p* < 0.05 compared to control cells treated with NH_4_Cl (d).

The appearance of cytoplasmic vacuolization in U251 cells treated with MD or AA+MD combination prompted us to investigate their ability to induce autophagy. TEM analysis at higher magnification confirmed the presence of autophagic vacuoles containing cytoplasmic material in both MD- and AA+MD-treated U251 cells (Figure 3(a)). Furthermore, MD alone and especially in combination with AA significantly increased the fluorescence of LysoTracker-stained cells, indicating an increase in the number/volume of acidic vacuoles, such as autolysosomes and/or lysosomes (Figure 3(b)). MD alone also increased the levels of proautophagic protein beclin-1, as well as autophagosome associated LC3-II, while reducing the protein levels of p62, a specific substrate for autophagic proteolysis (Figure 3(c)). These effects were more pronounced in combined treatment, particularly at the earlier time point (1 h), implying an accelerated/increased autophagic turnover in AA+MD-exposed cells. While usually resulting from the increase in the generation of autophagosomes, accumulation of LC3-II may also reflect the inhibition of autophagic proteolysis of LC3-II [41]. This is demonstrated by the increase in LC3-II levels upon incubation with the saturating concentration (defined in preliminary experiments) of lysosomal inhibitor NH_4_Cl, which blocks autophagic proteolysis (Figure 3(d)). However, the treatment with MD, both alone and especially in combination with AA, further increased NH_4_Cl-induced accumulation of LC3-II, indicating a genuine increase in autophagic flux (Figure 3(d)). In addition to MD-triggered upregulation of mRNA encoding *beclin-1* and *GABARAP* (mammalian homologs of ATG6 and ATG8, respectively), RT-qPCR analysis demonstrated the ability of combined treatment to stimulate the expression of autophagy transcription factors *FOXO1* and *FOXO3*, as well as proautophagic regulators *ATG13, BIF*, and *UVRAG* (Figure 3(e)). Thus, AA enhances autophagy gene transcription and autophagic flux in MD-treated U251 cells.

### 3.4. AA+MD Combination Induces Autophagy via mTOR Inhibition, ULK1 Activation, and ROS Production

**Figure 4:**
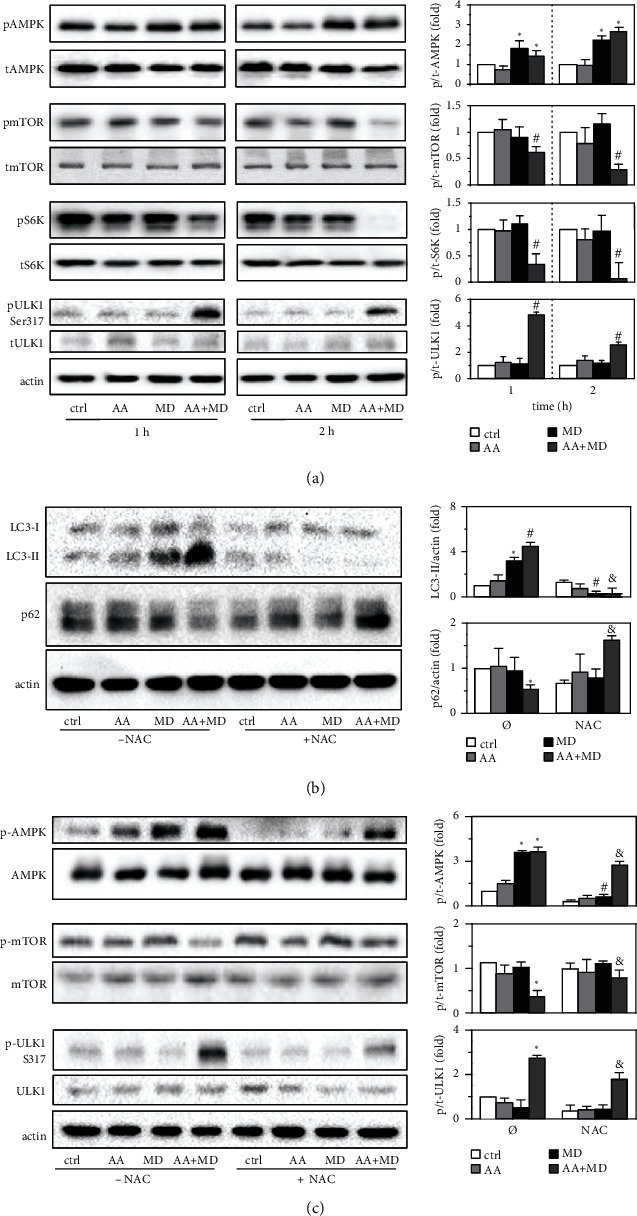
AA+MD induces ROS-mediated AMPK/mTOR/ULK1-dependent autophagy in U251 cells. U251 cells were incubated with AA (1 mM), MD (20 *μ*M), and AA+MD for 1 and 2 h (a) in absence or presence of NAC (2 mM) (b, c). The levels of phospho-AMPK (p-AMPK), AMPK, phospho-mTOR (p-mTOR), mTOR, phospho-S6K (p-S6K), S6K, phospho-ULK1 (Ser317), ULK1, and actin as a loading control were analyzed by immunoblotting and quantified using densitometry. The representative immunoblots are shown, while densitometry data (relative to total protein level) are presented as mean ± SD values from three independent experiments. ^∗^*p* < 0.05 compared to untreated, control cells in which the protein expression was arbitrarily set to 1; ^#^*p* < 0.05 compared to treatment with MD alone; ^&^*p* < 0.05 compared to control cells treated with AA+MD (b, c).

We next investigated the involvement of AMPK/mTORC1 signaling pathway and ROS in MD- and AA+MD-mediated autophagy. As demonstrated by immunoblot analysis, both MD and combined treatment significantly increased the phosphorylation of the intracellular energy sensor AMPK (Figure 4(a)). Surprisingly, only the combined treatment reduced the phosphorylation of the main autophagy repressor mTORC1 and its target S6K and stimulated AMPK-dependent phosphorylation of autophagy initiator ULK1 at Ser317 (Figure 4(a)). Considering that ROS are an important stimulus for autophagy induction [42], and both MD and AA+MD are found to induce oxidative stress (Figures 2(f) and 2(g)), we also assessed the role of ROS in MD- and AA+MD-triggered autophagy. The immunoblot analysis revealed that antioxidant and a source of thiol groups NAC blocked both MD- and AA+MD-induced LC3 conversion and p62 degradation (Figure 4(b)). NAC also prevented AMPK stimulation induced by MD alone and in combination with AA, as well as ULK1 activation and mTORC1 inhibition upon combined treatment (Figure 4(c)).

### 3.5. Autophagy Contributes to AA+MD-Induced Necrotic Death of U251 Cells

**Figure 5:**
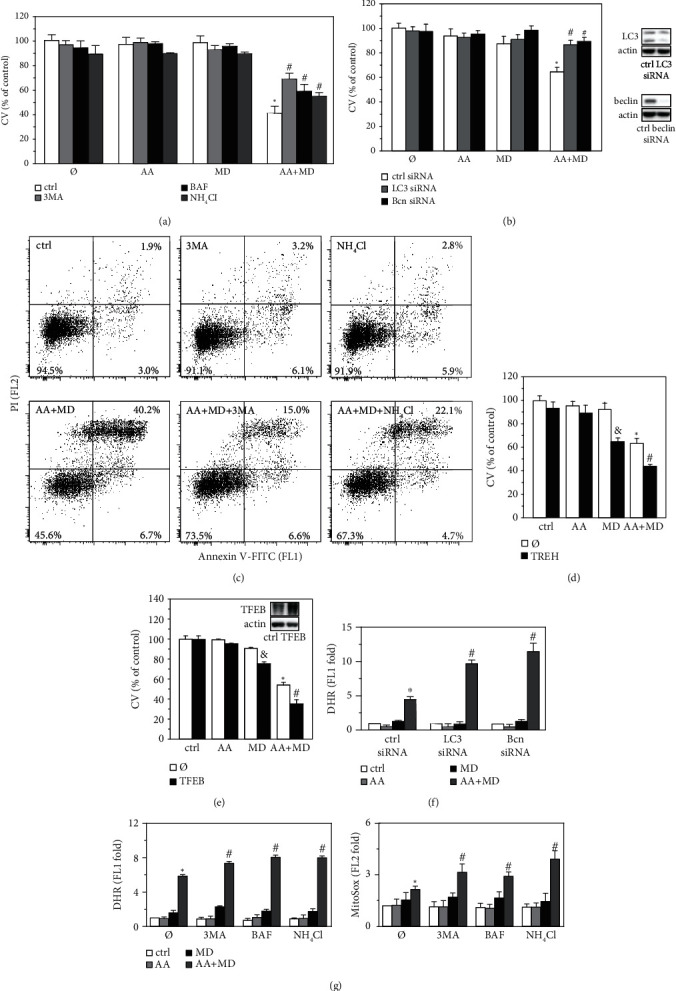
Autophagy induced by AA+MD has cytotoxic effect on U251 cells. Untransfected U251 cells (a, c, d, g), cells transfected with control, LC3, and beclin-1 siRNA (b, f), or cells transfected with control and TFEB-inserted plasmid vector (e) were treated with AA (1 mM), MD (20 *μ*M), or their combination (AA+MD) for 8 h (a) or 4 h (b–g) in presence or absence of 3MA (5 mM) (a, c, g), BAF (10 nM) (a, g), NH_4_Cl (12 mM) (a, c, g), or trehalose (TREH; 50 mM) (d). The transfection efficiency was confirmed by immunoblot analysis of the LC3, beclin-1, and TFEB levels, and representative immunoblots are shown (b, e). AnnexinV-FITC/PI-stained cells were analyzed by using flow cytometry (c). The number of viable cells was determined by CV assay, and intracellular (f, g) and mitochondrial (f) ROS concentrations were determined by flow cytometry after staining cells with DHR (f, g) or MitoSox (g), respectively. Data are presented as mean ± SD values of triplicates from a representative of three independent experiments (a–b, d–g), or representative dot plots from one of three experiments (c). ^∗^*p* < 0.05 compared to untreated, control cells; ^#^*p* < 0.05 compared to control cells treated with AA+MD (a–b, d–g); ^&^*p* < 0.05 compared to control cells treated with MD (d, e).

Finally, we investigated the role of MD- and AA+MD-induced autophagy in U251 glioblastoma cell death. 3-methyladenine, an inhibitor of class III PI3K-dependent autophagosome formation, as well as lysosomal inhibitors of autophagy BAF and NH_4_Cl all partly recovered the viability of AA+MD-treated cells, while the viability of cells exposed to MD alone was not affected (Figure 5(a)). Accordingly, genetic suppression of autophagy by LC3 or beclin-1 siRNA restored the viability of AA+MD-treated cells to a certain extent, but failed to affect the cytotoxicity of MD alone (Figure 5(b)). Moreover, flow cytometric analysis demonstrated that 3-methyladenine and bafilomycin A1 reduced the cell membrane damage and ensuing necrotic cell death induced by combined treatment (Figure 5(c)). Autophagy induction with disaccharide trehalose [43] or by overexpression of autophagy transcription factor TFEB stimulated the cytotoxicity of both MD alone and AA+MD combination (Figures 5(d) and 5(e)). These results indicate that MD induces nontoxic autophagy, which becomes cytotoxic upon additional activation with AA. Since autophagy mainly serves as an antioxidative mechanism [42], we next evaluated the effect of MD- and AA+MD-stimulated autophagy on ROS production. RNAi-mediated knockdown of beclin-1 or LC3 amplified ROS production upon combined treatment, without affecting ROS levels in cells treated with MD alone (Figure 5(f)). Accordingly, 3-methyladenine, bafilomycin A1, and NH_4_Cl stimulated the accumulation of ROS and mitochondrial superoxide in cells exposed to combined treatment, but not in cells treated with MD alone (Figure 5(g)), suggesting the involvement of autophagy in ROS clearance in combination treated cells. Therefore, while autophagy contributes to necrotic cell death induced by AA+MD combination, this effect does not depend on autophagy-mediated modulation of oxidative stress.

## 4. Discussion

In the present study, we demonstrate the ability of AA and MD, at subtoxic doses when administrated alone, to synergize in ROS- and mitochondrial membrane depolarization-mediated necrotic death of human U251 glioblastoma cells. Moreover, while mild oxidative stress induced by MD alone triggers AMPK activation and nontoxic autophagy, excessive ROS accumulation by combined treatment induces cytotoxic autophagy associated with mTORC1 inhibition and ULK1 activation.

The cytotoxic effect of AA+MD combination against U251 cells in the present study is consistent with the previously shown toxicity of this treatment in different glioblastoma cell lines, as well as in patient-derived glioblastoma cells [12, 44]. As previously shown in different types of malignant cells [10, 13, 45], including glioblastoma cell line [12], the redox cycling between ascorbate and menadione leads to excessive generation of ROS. Indeed, although AA itself did not induce oxidative stress, it stimulated ROS production triggered by MD in U251 cells. In line with the ability of oxidative stress to induce dissipation of mitochondrial potential [38, 46], extremely high production of ROS upon combined treatment was associated with mitochondrial depolarization. Oxidative stress and mitochondrial depolarization are the main mediators of both apoptotic [47] and necrotic cell death [48]. However, our results showed cell membrane damage in the absence of caspase/PARP activation and Ann^+^/PI^−^ apoptotic cells, indicating that AA+MD combination mainly induced necrosis-like death of U251 cells. The inability of combined treatment to induce energy-dependent apoptotic cell death [49] could be at least partly explained by the previously shown capacity of MD to inhibit oxidative phosphorylation [8, 9], as well as by the loss of caspase function in a highly oxidative environment [50]. Accordingly, caspase-3/PARP-independent cell death has already been reported in AA+MD-treated hepatocarcinoma and erythromyeloid leukemia cells [13, 51]. It can be assumed that mitochondrial impairment and presumable cell energy pool discharge triggered by the AA+MD would especially target oxidative phosphorylation-dependent GBM cells, which are accountable for recurrence and therapeutic resistance of tumor [52]. In addition, glycolysis-dependent GBM cells can readily recruit mitochondrial pathways for growth and survival in response to glucose deficiency [53]. Previous poses an interesting opportunity that mitochondrial function impairment by AA+MD can ensure the potential of the treatment to eradicate resistance-prone cells and/or highly adaptable bulk GBM cells.

The key role of oxidative stress in AA+MD toxicity towards U251 glioblastoma cells was confirmed by the cytoprotective effect of antioxidant NAC and is in line with other studies [10, 11]. The observed NAC potential to oppose AA+MD cytotoxicity could be attributed to its ability to enhance the reducing power of the cells through replenishing intracellular glutathione (GSH) levels [54], since several studies till date refer that cytotoxicity of both MD [7] and AA+MD [12, 55] significantly relies on GSH depletion in cancer cells. This is in line with the capacity of other thiol group sources such as L-cysteine and dithiothreitol to diminish the toxicity of MD in breast and glioblastoma cancer cells [56, 57]. However, NAC also prevented both LC3 conversion and p62 degradation induced by MD and AA+MD, confirming that ROS generation triggered by these treatments was responsible for autophagy induction. These results are in accordance with the well-known role of oxidative stress as an autophagy inducer [50]. Higher ROS production induced by AA+MD combination, compared to MD alone, correlated with an augmented autophagic response and enhanced autophagic flux, as judged by the increase in beclin-1 expression, p62 degradation, and LC3 conversion in the presence of lysosomal inhibitor. Moreover, combined treatment significantly increased the expression of eight, while MD upregulated only two out of eleven ATG genes tested. The genes upregulated by AA+MD combination involved those encoding autophagy transcription factors FOXO1/3 [41], as well as the members of autophagy initiation complex (beclin-1, ATG13, BIF1, and UVRAG) and molecules involved in autophagosome elongation (GABARAP). Whether this transcriptional activation indicates that excessive ROS generation is involved in the induction of nonselective autophagy at the expense of its selective forms, as some authors speculate [58], or simply reflects the higher proautophagic input, remains to be further investigated.

While both MD alone [25–32] and its combination with AA [10, 15, 33] have been found to induce autophagy in various types of cancer cells, the present study indicates the involvement of AMPK/mTORC1 signaling pathway in this process in the human U251 glioblastoma cell line. In accordance with previous findings obtained in leukemia cells [59], prostate carcinoma cells [60], and kidney cells of young rats [61], as well as with MD capacity to deplete energy [8, 9], we demonstrated that MD increased the activation of the energy sensor AMPK in U251 cells. However, in contrast to the previously shown ability of AA to mitigate MD-induced AMPK activation in leukemia cells [59], it had no significant effect on MD-mediated AMPK phosphorylation in our study. This discrepancy could be at least in part explained by cell type-specific responses and/or differences in drug concentrations. Although not directly confirmed, the role of AMPK in MD- and AA+MD-induced autophagy is indicated by the ability of NAC to suppress both AMPK and autophagic responses. Furthermore, we demonstrated potent mTOR inhibition concurrently with AA+MD-induced GBM cell death, which is in line with the results of Ivanova et al. [62]. This study showed the high synergistic toxic effect of low doses of AA+MD in combination with everolimus, a well-known mTOR inhibitor, toward leukemia cancer cells, suggesting a significant impact of mTOR inhibition on AA+MD cytotoxic potential. The capacity of AA+MD and everolimus to induce autophagy was not evaluated in the abovementioned study [62] and therefore it cannot be concluded if augmentation of AA+MD cytotoxicity by mTOR inhibition is related to autophagy activation solely or involves some other autophagy-independent mechanisms.

Costimulation of MD-treated cells with AA and a subsequent increase in ROS generation resulted in a qualitatively different signaling response including mTORC1/S6K inhibition and ULK1 activation. The activity of AMPK, ULK1, and mTORC1 is closely connected, as AMPK directly stimulates ULK1 activation by phosphorylation on Ser317 [63], while mTORC1 prevents AMPK/ULK1 interaction by phosphorylating ULK1 at Ser757 [63]. With this in mind, we suggest that activation of AMPK by MD was insufficient to stimulate ULK1 in the presence of active mTORC1, but readily activated ULK1 upon cotreatment with AA and subsequent suppression of mTORC1. However, it remains to be explained why MD-activated AMPK failed to inhibit mTORC1, and which signaling mechanisms were responsible for mTORC1 supression by combined treatment. Interestingly, it has been shown that ULK1 can inhibit mTORC1 [64], thus raising the possibility that mTORC1 inhibition by AA+MD combination was actually secondary to ULK1 activation. Regardless of the underlying mechanisms, inhibition of mTORC1 activity by combined treatment may be particularly important given the significant role of the overactivated PTEN/PI3K/AKT/mTORC1 pathway in glioblastoma initiation and progression [65].

Possibly, the most intriguing finding of our study is that cotreatment with AA converted MD-induced nontoxic autophagic response into cytotoxic autophagy, which contributed to necrotic cell death induced by the combined treatment. These data seem to contradict previous results, which, with one exception [29], demonstrated the cytoprotective effect of MD- or AA+MD-triggered autophagy, mediated by clearing misfolded proteins, maintaining DNA integrity, and/or replenishing ATP levels [25, 26, 31, 33]. In another study, AA+MD-induced autophagy apparently did not influence coinciding cell death [10]. These discrepancies could be at least partly explained by cell type-specific responses and/or differences in AA+MD concentrations and other experimental conditions. However, it should be noted that the studies demonstrating the protective or no role of autophagy in AA+MD-triggered cancer cell death relied solely on pharmacological agents such as 3-methyladenine or chloroquine to block autophagosome formation or autophagic proteolysis, respectively [10, 33]. Neither agent is a specific autophagy inhibitor, with 3-methyladenine even being able to induce autophagy in certain conditions [36], and chloroquine displaying autophagy-independent cytotoxicity [34, 35]. In addition to pharmacological inhibitors, we used genetic knockdown of crucial autophagy regulators beclin-1 and LC3 to show that AA+MD-triggered oxidative stress was associated with the induction of cytotoxic autophagy. Further, pharmacological and genetic suppression of autophagy increased the production of ROS induced by AA+MD combination, indicating that autophagy acts downstream, rather than upstream of ROS in inducing U251 cell death in our experimental system. Indeed, ROS have been implicated in mTOR inhibition-dependent cytotoxic autophagy in different GBM cell lines treated with various anticancer drugs [66–69]. Therefore, it could be speculated that increasing autophagy above a certain threshold through excessive ROS generation and subsequent modulation of AMPK/mTOR/ULK1 pathway is responsible for the synergistic cytotoxicity of AA+MD combination in U251 glioblastoma cells. Such an assumption is supported by the data showing that additional autophagy stimulation with trehalose or TFEB expression also converts MD-induced autophagy from nontoxic to cytotoxic. A similar switch from cytoprotective to cytotoxic autophagy has been described in irradiatied breast cancer cells additionally exposed to vitamin D3 [70]. However, the possibility that AA triggered some autophagy-independent events that synergized with the ongoing autophagy to cause cytotoxicity cannot be excluded and is worthy of further consideration.

Numerous studies show that AA+MD combination selectively targets various cancer cells, including GBM [16, 71], over the nontransformed cells of the same origin [11, 17, 40, 45, 55, 62, 72]. The ability of the combination to preferentially damage cancer cells is attributed to the strong overproduction of mitochondrial superoxide due to MD and AA redox cycling in dysfunctional/overcharged mitochondria, which is typical for cancer cells. As a consequence of strong ROS generation, the complex disturbance of energy substrates (ATP, NADH/NAD^+^) and a decrease in levels of major oncometabolite succinate [16, 40] appears only in cancer cells. Besides, ascorbate is selectively taken up by cancer cells, including GBM [73], due to overexpression of glucose transporters GLUT1, SVCT1, and SVCT2 [74], thus enabling vigorous in situ ascorbate-driven menadione redox cycling in transformed cells. On the other hand, menadione prenylation to vitamin K2 decreases MD levels exclusively in normal cells [75] and subsequently prevents AA+MD-induced oxidative stress and cell death [55]. The low levels of antioxidative enzymes like catalase [17] and glutathione peroxidase [12] could contribute to the greater sensitivity of cancer cells to AA+MD-driven oxidative insult, too. Taking the abovementioned into account, we can assume that selective and enhanced production of ROS in cancer, but not in nontransformed cells, would presumably force autophagy into a cytotoxic direction only in GBM cells. The latter has a foothold in the previous studies that reported opposed endpoints of ROS-mediated autophagy in GBM and related nontransformed cells [76] or different capacities of cancer and healthy cells challenged by diverse ROS generating agents to activate autophagy [77, 78]. The study by Matteoni et al. [76] demonstrated the potential of chlorpromazine, a well-known neuroleptic drug, to concurrently trigger cytotoxic autophagy in T98G, U-251, and U-87 GBM cells and cytoprotective autophagy in noncancer RPE-1 epithelial cells. Next, several ROS-generating agents (H_2_O_2_, 2-methoxyestradiol, and mitochondrial electron transport chain inhibitors) caused autophagy-mediated cell death in U87 GBM cells, but failed to significantly affect redox homeostasis and induce autophagy in primary mouse astrocytes [77, 78]. Overall, data presented in this paper, together with the results outlined in the referred studies, advocate for a significant advantage of AA+MD application in GBM therapy.

## 5. Conclusions

**Figure 6:**
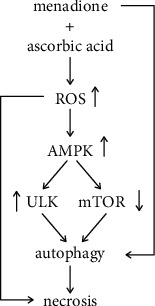
A hypothetical model of AA+MD-mediated antiglioblastoma action in U251 cells.

In conclusion, the present study demonstrates that ROS-dependent modulation of mTORC1 and ULK1 activity triggers an excessive autophagic response that contributes to the synergistic induction of necrotic death in U251 cells exposed to a combination of AA and MD (Figure 6). While the molecular mechanisms of the observed effects remain to be fully clarified, the finding that additional stimulation of autophagy increases the cytotoxicity of both MD alone and its combination with AA could be employed in GBM therapy. Such an assumption is supported by the ability of AA and MD to cross the blood brain barrier [73], as well as by the documented safety of the combined treatment in human studies [18]. Although one must be careful in interpreting data obtained using a single cell line, the revealed molecular mechanisms give a sound basis for the development of therapeutic strategy which could circumvent the resistance of GBM to conventional chemotherapeutics.

## Data Availability

All relevant data used to support the findings of this study are included within the article.

## Abbreviations

MD: Menadione
AA: Ascorbic acid
3MA: 3-methyladenine
BAF: Bafilomycin A1
TREH: Trehalose
NAC: N-acetylcysteine
ROS: Reactive oxygen species
mTORC1: Mechanistic target of rapamycin complex 1
ULK1: Unc-51-like autophagy-activating kinase 1
AMPK: AMP-activated protein kinase
LC3B: Microtubule-associated protein 1 light-chain 3B
PARP: Poly (ADP-ribose) polymerase-1
TFEB: Transcription factor EB
DHR: Dihydrorhodamine 123
JC-1: 5,5′,6,6′-tetrachloro 1,1′,3,3′-tetraethylbenzimidazolocarbocyanine iodide
LTR: LysoTracker red DND-99.
